# 

**Published:** 1887-10

**Authors:** 


### CONTENTS.

### COMMUNICATIONS.

Ninth International Medical Congress.......... 473
Facial Neuralgia.......... 484
Chronic Pyæmia of Dental Origin.......... 487
A New Method of Producing Local Anæsthesia of the Skin.......... 504
Simple Fracture of the Lower Jaw.......... 509

### SELECTIONS.

Hints on Some Points in Eye Diseases Occurring in Children.......... 512
Brown Bread.......... 516
British Medical Temperance Association.......... 517
Hare-Lip Operation.......... 517
Science of Hypnotism.......... 519

### EDITORIAL.

Educational.......... 520
Medical Congress—Dental Section.......... 521
Method for Deodorizing Iodoform.......... 524
Ohio State Dental Society.......... 524

